# Correction: The coenzyme A precursor pantethine enhances antitumor immunity in sarcoma

**DOI:** 10.26508/lsa.202302479

**Published:** 2023-11-29

**Authors:** Richard Miallot, Virginie Millet, Anais Roger, Romain Fenouil, Catherine Tardivel, Jean-Charles Martin, Fabrice Tranchida, Laetitia Shintu, Paul Berchard, Juliane Sousa Lanza, Bernard Malissen, Sandrine Henri, Sophie Ugolini, Aurélie Dutour, Pascal Finetti, François Bertucci, Jean-Yves Blay, Franck Galland, Philippe Naquet

**Affiliations:** 1 INSERM, CNRS, Centre D’Immunologie de Marseille-Luminy , Aix-Marseille Université, Marseille, France; 2 INRAE, INSERM, C2VN, Aix Marseille Université, Marseille, France; 3 CNRS, Centrale Marseille, ISM2, Aix Marseille Université, Marseille, France; 4 INSERM 1052, CNRS 5286, Cancer Research Center of Lyon (CRCL), Childhood Cancers and Cell Death Laboratory, Lyon, France; 5 INSERM, CNRS, Centre D’Immunophénomique (CIPHE), Aix Marseille Université, Marseille, France; 6 INSERM, CNRS, Centre de Recherche en Cancérologie de Marseille (CRCM), Institut Paoli-Calmettes (IPC), Laboratory of Predictive Oncology, Aix-Marseille Université, Marseille, France; 7 Department of Medical Oncology, Institut Paoli-Calmettes, Marseille, France; 8 UNICANCER Centre Léon Bérard, Department of Medicine, Université Lyon I, Lyon, France

## Abstract

VitB5 level becomes limiting in sarcomas. It is regulated by the pantetheinase activity of VNN1. VNN1 expression in sarcomas is associated with better prognosis and immune infiltration. In mice, complementation with pantethine, a vitB5 precursor, boosts antitumor immunity.

Article: Miallot R, Millet V, Roger A, Fenouil R, Tardivel C, Martin J-C, Tranchida F, Shintu L, Berchard P, Sousa Lanza J, Malissen B, Henri S, Ugolini S, Dutour A, Finetti P, Bertucci F, Blay J-Y, Galland F, Naquet P (2023 Oct 13) The coenzyme A precursor pantethine enhances anti-tumor immunity in sarcoma. Life Sci Alliance 6(12): e202302200. doi: 10.26508/lsa.202302200. PMID: 37833072.

This revised version of our article adds Fabrice TRANCHIDA as a coauthor. His name was inadvertently omitted from the original version. Fabrice Tranchida performed the analysis of the NMR data.

## Supplementary Material

Reviewer comments

