# Supplementary material for: Correction: The coenzyme A precursor pantethine enhances antitumor immunity in sarcoma
Source: Life Sci Alliance. 2023 Nov 29;7(2):e202302479. doi: 10.26508/lsa.202302479 (PMC10690990; doi:10.26508/lsa.202302479)
Supplement: Reviewer comments [file LSA-2023-02479_review_history.pdf]

Life Science Alliance

Correction: The coenzyme A precursor pantethine enhances anti-tumor immunity in sarcoma

Philippe Naquet
DOI: https://doi.org/10.26508/lsa.202302479

Corresponding author(s): Philippe Naquet, Centre d'Immunologie de Marseille-Luminy

**Review Timeline:**	Submission Date:	2023-11-13
	Accepted:	2023-11-14

Scientific Editor: Eric Sawey, PhD

Transaction Report:

(Note: With the exception of the correction of typographical or spelling errors that could be a source of ambiguity, letters and reports are not edited. The original formatting of letters and referee reports may not be reflected in this compilation.)

November 13, 2023

RE: Life Science Alliance Manuscript #LSA-2023-02479

Prof. Philippe Naquet
Centre d'Immunologie de Marseille-Luminy
CIML
INSERM-CNRS-Univ. Méditerranée Case 906 Cedex 9
Marseille, PACA 13288
France

Dear Dr. Naquet,

Thank you for submitting your Correction entitled "Correction: The coenzyme A precursor pantethine enhances anti-tumor immunity in sarcoma".

IMPORTANT: If you will be unreachable at any time, please provide us with the email address of an alternate author. Failure to respond to routine queries may lead to unavoidable delays in publication.

Scheduling details will be available from our production department.

You can contact the journal office with any questions, contact@life-science-alliance.org

Sincerely,

Eric Sawey, PhD
Executive Editor
Life Science Alliance
http://www.lsajournal.org

November 13, 2023

RE: Life Science Alliance Manuscript #LSA-2023-02479

Prof. Philippe Naquet
Centre d'Immunologie de Marseille-Luminy
CIML
INSERM-CNRS-Univ. Méditerranée Case 906 Cedex 9
Marseille, PACA 13288
France

Dear Dr. Naquet,

Thank you for submitting your Correction entitled "Correction: The coenzyme A precursor pantethine enhances anti-tumor immunity in sarcoma".

IMPORTANT: If you will be unreachable at any time, please provide us with the email address of an alternate author. Failure to respond to routine queries may lead to unavoidable delays in publication.

Scheduling details will be available from our production department.

You can contact the journal office with any questions, contact@life-science-alliance.org

Sincerely,

Eric Sawey, PhD
Executive Editor
Life Science Alliance
http://www.lsajournal.org
